# New insights into a sensitive life stage: hydraulics of tree seedlings in their first growing season

**DOI:** 10.1111/nph.20243

**Published:** 2024-11-05

**Authors:** Barbara Beikircher, Magdalena Held, Adriano Losso, Stefan Mayr

**Affiliations:** ^1^ Department of Botany Universität Innsbruck/University of Innsbruck Sternwartestr. 15 Innsbruck 6020 Austria; ^2^ Institute for Atmospheric and Earth System Research (INAR)/Forest Sciences University of Helsinki PO Box 64 Helsinki 00014 Finland

**Keywords:** drought resistance, embolism, hydraulic efficiency, hydraulic safety, seedling, tree hydraulics, ultrasonic

## Abstract

- The first year in a tree's life is characterized by distinct morphological changes, requiring constant adjustments of the hydraulic system. Despite their importance for the natural regeneration of forests and future vegetation composition, little has been known about the hydraulics of tree seedlings.
- At different times across the first growing season, we analysed xylem area‐specific (*K*_shoot_Axyl_) and leaf area‐specific (*K*_shoot_L_) shoot hydraulic conductance, as well as embolism resistance of three temperate conifer trees, two angiosperm trees and one angiosperm shrub, and related findings to cell osmotic parameters and xylem anatomical traits.
- Over the first 10 wk after germination, *K*_shoot_Axyl_ and *K*_shoot_L_ sharply decreased, then remained stable until the end of the growing season. Embolism resistance was remarkably low in the youngest stages but, coupled with an increase in cell wall reinforcement, significantly increased towards autumn. Contemporaneously, water potential at turgor loss and osmotic potential at saturation decreased.
- Independent of lineage, species and growth form, the transition from primary to secondary xylem resulted in a less efficient but increasingly more embolism‐resistant hydraulic system, enabling stable water supply under increasing risk for low water potentials.

## Introduction

The seedling stage is a special and the most critical one in a tree's life: No other life phase is subjected to such rapid morphological and, therewith related, physiological changes. Within few months, plants develop from small, seed resource‐dependent and highly vulnerable seedlings to self‐sustaining and more resilient juvenile plants (Facelli, 2008; Reinhardt *et al*., 2015; Miller & Johnson, 2017; Augustine & Reinhardt, 2019; Brodersen *et al*., 2019; Miller *et al*., 2020; Beikircher *et al*., 2021). In the present study, the term ‘seedling’ is strictly used for plants in their first year, while in the literature also more (e.g. plants with cotyledons only) or less (e.g. several‐year‐old plants) restrictive definitions are found (Fenner, 1987; Johnson *et al*., 2011; Brodersen *et al*., 2019; Martini, 2023). Furthermore, we use the term ‘seedling establishment’ for the entire first growing season, while other studies defined it shorter (e.g. the period between germination and shedding of cotyledons) or longer (i.e. beyond the first year; also see Fenner & Thompson, 2005; Leck *et al*., 2008; Brodersen *et al*., 2019).

For successful seedling establishment, the development of an efficient and adequately safe hydraulic system is certainly of utmost importance. Initially relying entirely on reserves stored in seeds (Leck & Outred, 2008), maximizing photosynthesis is of top priority to enable growth, which is crucial to access soil water‐ and light resources, which in turn improves competitiveness and increases the likelihood of survival (Sack, 2004; Fenner & Thompson, 2005; Facelli, 2008; Bebre *et al*., 2021). Accordingly, primary leaves of several species have higher photosynthetic capacity than mature foliage (Bond, 2000). However, for optimal photosynthesis, stem and leaf hydraulic conductance (*K*) also needs to be sufficiently high (Brodribb & Feild, 2000; Hubbard *et al*., 2001; Mencuccini, 2003; Brodribb *et al*., 2017). Many studies have shown a tight hydraulic‐stomatal coordination across species and at different plant levels (roots, stems and leaves) for at least several‐year‐old plants (Mencuccini, 2003; Sack & Holbrook, 2006; Charra‐Vaskou & Mayr, 2011; Way *et al*., 2014; Scoffoni *et al*., 2016; Brodribb *et al*., 2017). Strong correlations between stomatal and shoot (Beikircher *et al*., 2021) or root (Hernández *et al*., 2010) *K* have also been found in tree seedlings, and Zhong *et al*. (2020) reported a remarkable co‐variation in xylem and anatomical traits across 53 European woody angiosperm seedlings. However, Johnson *et al*. (2011), Miller & Johnson (2017) and Beikircher *et al*. (2021) have also observed an ontogenetic decoupling of xylem and leaf traits *c*. 1 month after germination in some angiosperm and conifer species: At a certain point between 3 and 10 wk after germination, the increase in hydraulic efficiency (i.e. xylem area‐specific shoot conductance) lagged behind the increase in leaf area (LA) and thus the hydraulic sufficiency (i.e. LA‐specific conductance; Tyree & Zimmermann, 2002) declined.

In general, higher *K* can be achieved by developing more, wider and/or longer conduits with low pit resistance (Becker *et al*., 1999; Mencuccini, 2003; Sack *et al*., 2003; Sperry *et al*., 2006; Choat *et al*., 2008). As tree seedlings start out with only few primary xylem conduits, those need to be highly conductive to ensure sufficient water supply to leaves. Primary xylem elements are often larger in diameter and thus potentially more conductive than secondary xylem conduits (Tyree & Zimmermann, 2002; Miller *et al*., 2020), but also more susceptible to drought‐induced embolism (Choat *et al*., 2005, 2015; Venturas *et al*., 2019; Miller *et al*., 2020; Haverroth *et al*., 2024). However, due to a lack of studies, it remains unknown how morphological, structural and functional traits change from primary to secondary xylem.

Little is also known about hydraulic safety, that is the ability of the xylem to prevent the formation of xylem embolism, of developing seedlings. Drought‐induced embolism occurs when the evaporative demand exceeds supply and xylem pressure decreases below species‐specific thresholds (Tyree & Zimmermann, 2002). Air is then sucked into water‐filled conduits via pits from adjacent air‐filled areas (Sperry & Tyree, 1988; Mayr *et al*., 2014). Consequently, pit anatomy plays a major role in preventing air‐seeding and defining a plant's hydraulic safety (Hacke & Sperry, 2001; Choat *et al*., 2008; Kaack *et al*., 2019). The cell wall reinforcement, that is conduit wall thickness to span ratio ((*t*/*b*)^2^), also plays a major role, as the higher the ratio, the higher the embolism resistance (Hacke & Sperry, 2001). In the last decades, numerous studies have focused on embolism resistance between (Cochard, 1992; Charra‐Vaskou *et al*., 2012; Choat *et al*., 2012; Ganthaler & Mayr, 2015) and within (Beikircher & Mayr, 2009; Martinez‐Vilalta *et al*., 2009; Losso *et al*., 2016; Beikircher *et al*., 2019) species, as well as between organs of the same specimens (Tsuda & Tyree, 1997; Johnson *et al*., 2016; Ganthaler *et al*., 2019). Due to their small root system and limited internal water buffers, seedlings are particularly prone to drought, which is indeed the primary cause of seedling death in many ecosystems (Cui & Smith, 1991; Moles & Westoby, 2004; Engelbrecht *et al*., 2005; Facelli, 2008; Johnson *et al*., 2011; Reinhardt *et al*., 2015; Losso *et al*., 2018; Augustine & Reinhardt, 2019). Nevertheless, there is only limited information on hydraulic safety of seedlings. Only few studies have analysed embolism resistance in 4‐ to 12‐month‐old plants (Tsuda & Tyree, 1997; Lauenstein *et al*., 2013; Way *et al*., 2013; Losso *et al*., 2018). For less than 4‐month‐old plants, to our knowledge, only (Miller & Johnson, 2017; Miller *et al*., 2020) have performed corresponding analyses. In three conifers, the authors have found primary conduits to embolize already at water potentials above −1 MPa, while in the secondary xylem, embolism occurred well below −2 MPa. This has been explained by the observed decrease in torus thickness and increase in torus‐to‐pit overlap as well as cell reinforcement in secondary conduits, both of which are connected with higher embolism resistance (see Hacke *et al*., 2001; Bouche *et al*., 2014).

The paucity of studies on seedling hydraulics, especially on the youngest stages, is surprising given the importance of seedling survival for natural regeneration, and consequently for the survival and distribution of plant species and even entire forests. Moreover, in view of climate change and expected increase in drought events, seedling mortality will be a relevant aspect of future ecosystem development (Moles & Westoby, 2004; Anderegg & Anderegg, 2013; Kerr *et al*., 2015; Clark *et al*., 2016; Martínez‐Vilalta & Lloret, 2016; Augustine & Reinhardt, 2019; Brodersen *et al*., 2019). However, hydraulic studies on the youngest tree stages are challenging as many standard hydraulic methods are not applicable on tiny and fragile seedlings (Miller *et al*., 2020; Beikircher *et al*., 2021). All of the above‐mentioned hydraulic studies on seedlings younger than 4 months old were limited to three conifers and two angiosperms and, with the exception of Beikircher *et al*. (2021), datasets were either snapshots of a specific age or monitoring was stopped no later than 10 wk after germination. In addition, most of the datasets are based on theoretical calculations rather than actual measurements.

The purpose of the present study was to monitor changes in various hydraulic and related anatomical parameters in the xylem of seedlings between their germination in spring and the end of the first growing season in late autumn. The usage of five (widespread and economically important) European conifer (*Picea abies*, *Pinus sylvestris* and *Larix decidua*) and angiosperm (*Acer pseudoplatanus* and *Fagus sylvatica*) trees and one angiosperm shrub (*Viburnum lantana*) allowed to consider structure–function relationships among different taxa and growth forms. We hypothesized that morphology and hydraulics are tightly linked across ontogeny, whereby in early life stages hydraulic efficiency is prioritized over embolism resistance. In tight correlation with latter, we also anticipated cell osmotic adjustments and therewith related a general increase in drought tolerance. We expected ontogenetic changes to be highly species‐specific and driven by lineage (conifers vs angiosperms) and potentially growth form (e.g. trees vs shrubs) related differences.

## Materials and Methods

### Plant material and experimental design

Measurements were made on 3‐ to 29‐wk‐old seedlings of six woody species native to Central Europe: *Picea abies* (L.) H. Karst. and *Pinus sylvestris* L. are evergreen conifer trees, *Larix decidua* (Mill.) is a deciduous conifer tree, *Acer pseudoplatanus* L. and *Fagus sylvatica* L. are deciduous angiosperm trees and *Viburnum lantana* L. is a deciduous shrub.

In April 2016, cold stratified seeds were sown in pots filled with a mixture of soil designed for alpine plants (leaf mould : ground soil : coconut fibre : sand : horticultural lava : rock meal; 5 : 2 : 2 : 2 : 1 : 0.15). Upon emergence, plants were grown in a glasshouse in the Innsbruck Botanical Garden, Austria, and watered daily. Throughout the first growing season (i.e. April to October 2016), hydraulic and anatomical parameters specified below were analysed at intervals. Due to time constraints (measurements of a given cohort had to be carried out within few days) and different germination dynamics (e.g. *V. lantana* germinated 5 wk after *Acer* and *Fagus*), investigated age classes differed across species (Table 1). Also, *Fagus* was only analysed three times due to poor germination rates.

**Table 1:** Ecological and morphological characteristics of studied species and age classes, respectively.

Species and analysed age classes (weeks)	Plant type/Growth form/Successional stage	Plant height (cm)	Number of fully developed leaves	Total leaf area (LA; cm^2^)	Xylem area (*A*_xyl_; mm^2^)	Huber value (HV; m^2^ m^−2^ × 10^−6^)
*Picea abies*						
3	Evergreen conifer tree late stage	3.04 ± 0.14^a^	7–8 cotyledons	0.65 ± 0.04^a^	0.006 ± 0.001^a^	0.92 ± 0.16^a^
8		3.31 ± 0.30^ab^	–	1.35 ± 0.12^b^	0.031 ± 0.004^a^	2.30 ± 0.36^b^
12		4.06 ± 0.65^ab^	–	3.39 ± 0.41^bc^	0.115 ± 0.013^b^	3.39 ± 0.64^bc^
29		4.64 ± 0.28^b^	–	4.17 ± 1.09^c^	0.366 ± 0.012^c^	8.78 ± 2.34^c^
*Pinus sylvestris*						
3	Evergreen conifer tree early stage	2.77 ± 0.19^a^	6–7 cotyledons	0.56 ± 0.05^a^	0.007 ± 0.002^a^	1.25 ± 0.37^a^
8		3.16 ± 0.11^a^	–	1.77 ± 0.24^a^	0.029 ± 0.007^b^	1.64 ± 0.45^a^
12		6.56 ± 0.37^b^	–	6.66 ± 0.52^b^	0.225 ± 0.027^c^	3.38 ± 0.48^b^
29		9.10 ± 0.52^c^	–	9.63 ± 1.27^c^	0.665 ± 0.047^d^	6.91 ± 1.03^c^
*Larix decidua*						
3	Deciduous conifer tree early stage	3.43 ± 0.13^a^	6–7 cotyledons	0.51 ± 0.03^a^	0.003 ± 0.001^a^	0.59 ± 1.96^a^
8		3.53 ± 0.24^a^	–	1.41 ± 0.27^a^	0.042 ± 0.008^a^	2.98 ± 0.80^b^
12		6.54 ± 0.60^b^	–	6.87 ± 0.87^b^	0.172 ± 0.023^a^	2.50 ± 0.46^b^
22		22.49 ± 2.08^c^	–	28.68 ± 3.90^c^	1.193 ± 0.336^b^	4.16 ± 1.30^c^
*Acer pseudoplatanus*						
4	Deciduous angiosperm tree early to late stage	2.86 ± 0.15^a^	2 cotyledons	3.12 ± 0.18^a^	0.165 ± 0.018^a^	5.29 ± 0.65^a^
7		6.60 ± 0.65^b^	2	26.56 ± 2.90^b^	0.353 ± 0.058^a^	1.33 ± 0.26^b^
14		33.26 ± 3.01^c^	10–12	329.87 ± 36.90^c^	3.869 ± 0.310^b^	1.18 ± 0.16^b^
22		34.95 ± 2.52^c^	16–18	439.33 ± 43.96^c^	12.063 ± 1.792^c^	2.75 ± 0.49^b^
*Fagus sylvatica*						
4	Deciduous angiosperm tree late stage	2.79 ± 0.14^a^	2 cotyledons	8.38 ± 0.63^a^	0.173 ± 0.004^a^	2.06 ± 0.18^a^
9		9.54 ± 0.70^b^	2	53.40 ± 10.70^b^	0.274 ± 0.064^a^	0.51 ± 0.16^b^
22		12.88 ± 1.01^b^	5	64.75 ± 9.03^b^	2.663 ± 0.245^b^	4.11 ± 0.69^a^
*Viburnum lantana*						
4	Deciduous angiosperm shrub mid to late stage	1.78 ± 0.12^a^	2 cotyledons	1.71 ± 0.19^a^	0.006 ± 0.002^a^	0.35 ± 0.12^a^
6		3.84 ± 0.47^b^	2	10.46 ± 0.87^b^	0.071 ± 0.012^a^	0.68 ± 0.13^a^
8		5.72 ± 0.61^b^	4	31.88 ± 4.49^c^	0.353 ± 0.067^b^	1.11 ± 0.26^ab^
16		10.73 ± 0.59^c^	6–8	77.39 ± 7.50^d^	1.367 ± 0.026^c^	1.77 ± 0.17^b^

**Fig. 1:**
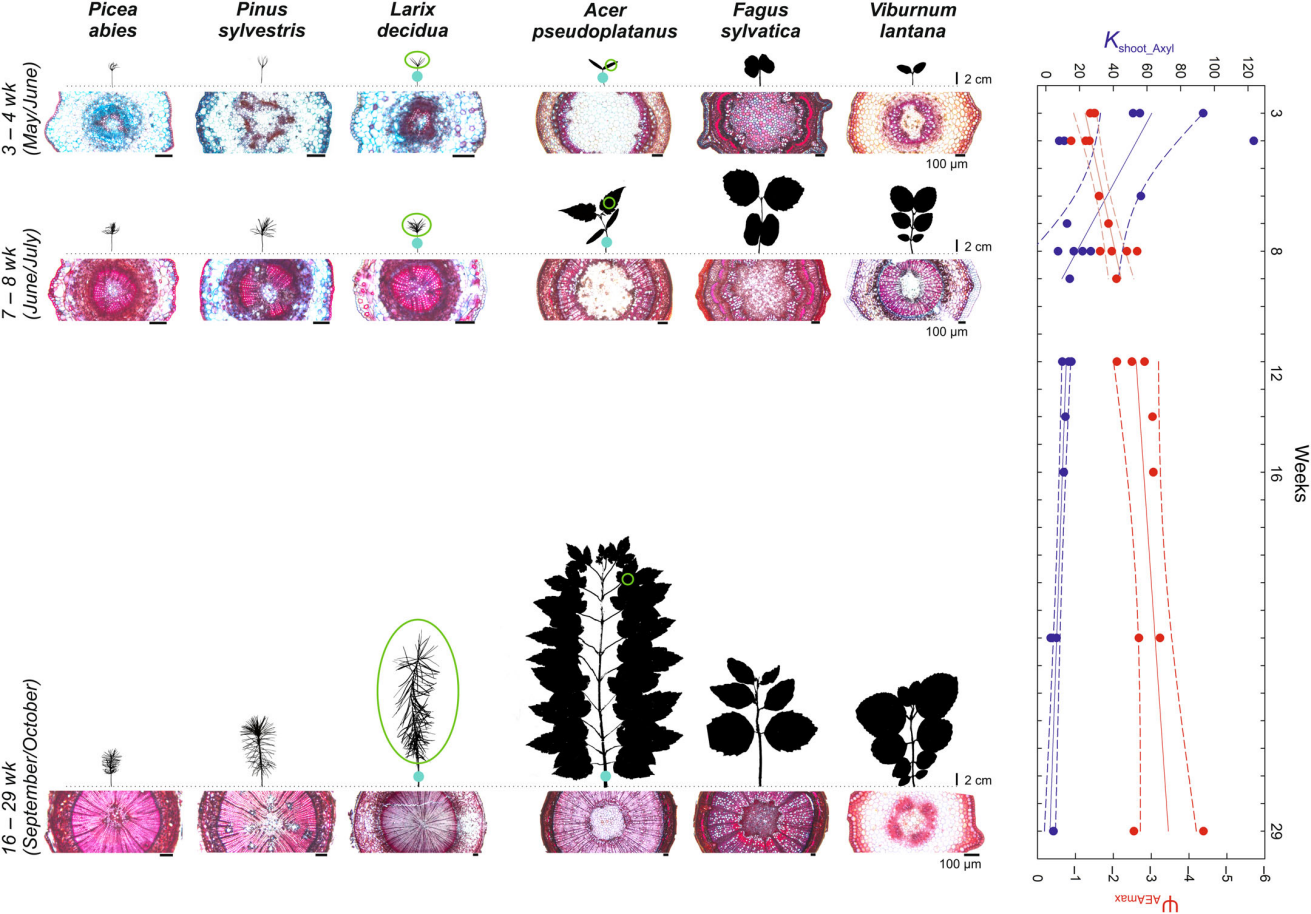
Schematic overview of selected age classes and respective phloroglucinol–hydrochloric acid stained cross sections through the hypocotyl. Shown are examples for youngest (i.e. 3‐ to 4‐wk‐old), 7‐ to 8‐wk‐old and oldest (i.e. 16‐ to 29‐wk‐old) seedlings. Green circles in the images of *Larix decidua* and Acer *pseudoplatanus* indicate exemplarily the parts of the plant from which samples for pressure–volume analyses were taken, blue circles indicate where ultrasonic sensors were attached and cross sections made. Please note that the age of plants at the end of the vegetation season (September/October) differs between species due to different germination times (for details see Material and Method section). Bars referring to seedling height equals 2 cm, bars for cross sections depict 100 μm. The right‐hand panel shows the xylem area‐specific conductance (*K*_shoot_Axyl_) and the water potential at maximum acoustic emission activity (Ψ_AEAmax_) across the season, with regression lines and 95% confidence intervals split into Weeks 3–8 and 12–29, respectively.

Mean ± SE. Different letters indicate significant differences (*P* < 0.05) across age classes within a given species. Please note that the number of leaves was only determined for angiosperm species. For an estimation of needle number in conifers, please see Fig. 1.

Before harvesting, plants were covered with dark plastic bags to stop transpiration for 30 min. Seedlings were then carefully dug out, placed in a beaker with water, covered again with a dark plastic bag and transported to the laboratory, where they were immediately analysed.

### Pressure–volume analysis

Water potential at turgor loss point (Ψ_TLP_; MPa), osmotic potential at saturation (Ψ_osat_; MPa) and modulus of elasticity (ε) were determined via pressure–volume analyses (Tyree & Hammel, 1972) on six plants per species and age class. For conifers, up to 10 needles per individual were selected randomly along the needle tuft and cut into *c*. 2‐mm‐long pieces. For angiosperms, one leaf disc per individual (6 mm in diameter) was cut with a cork borer from the penultimate fully developed leaf (Fig. 1). Samples were put in a sample holder, and turgid weights (TW) were immediately measured with an analytical balance (Sartorius BP61S, 0.0001 g precision; Sartorius AG, Göttingen, Germany). Samples were then sealed into C52‐psychrometers connected to a PsyPro datalogger (Wescor, Logan, UT, USA). After sufficient equilibration time (15–30 min), leaf water potential (Ψ_leaf_) was measured using the 50‐points array mode, which allows to (1) check whether settings were adequate to reach the wet bulb plateau, (2) ensure that μV‐readings at the plateau were used for automatic calculation of Ψ_leaf_, and (3) if not, to manually calculate Ψ_leaf_ from proper μV‐readings using the priorly obtained calibration equation of each C52‐psychrometer. Chambers were then opened to allow for further dehydration of the sample. In the following, fresh weight (FW) and respective Ψ_leaf_ were measured at intervals. After the last measurement, leaf samples were oven‐dried at 80°C for 24 h to obtain the dry weight (DW), and to allow for calculation of water saturation deficiency (WSD).

For analyses, always two samples were pooled to ensure sufficient data points per pressure–volume curve. Reciprocal Ψ_leaf_ was plotted vs WSD, and the turgescent section of the curve was fitted with a parabolic function while the osmotic section was fitted with a linear regression. For each pooled dataset Ψ_TLP_ (defined as the intersection of the parabolic and the linear regression), Ψ_o_ (defined as the intersection of the linear regression with the *y*‐axis) and ε (slope of the curve above Ψ_TLP_) were determined (Beikircher *et al*., 2019; Ganthaler *et al*., 2019), and values were then averaged per species and age class, respectively.

### Shoot hydraulic conductance

Hydraulic conductance of whole shoots was analysed on 5–10 individuals per species and age class, using the evaporative flux method (EFM; Sack *et al*., 2002). Following the procedure described by Beikircher *et al*. (2021), seedlings were cut under water at the hypocotyl base with a sharp razor knife and placed in a micro test tube (Eppendorf®) filled with flow solution, that is distilled, degassed and filtered (0.22 μm filter) water containing calcium chloride (CaCl_2_, 1 mmol) and potassium chloride (KCl, 10 mmol) to avoid bacterial growth and to standardize for potential ionic effects on K (see Nardini *et al*., 2012). To prevent water loss over the open water surface, the tube was tightly sealed with Parafilm® ‘M’ (Pechiney Plastic Packaging, Menasha, WI, USA) around the hypocotyl, and the Parafilm was perforated twice with a thin needle (0.5 mm diameter) to avoid decreasing air pressure in the tube during transpiration.

Shoots were then placed under a high intensity, fixed spectrum LED grow light (Heliospectra E602G, Gothenburg, Sweden) providing *c*. 1600 μmol m^−2^ s^−1^ of photosynthetically active radiation (measured with a PAR quantum sensor; Skye Instruments Ltd, Llandrindod Wells, UK) at leaf level for 20 min, to induce stomatal opening. To facilitate transpiration, a fan was positioned *c*. 30 cm aside the plant. Temperature and relative humidity (continuously monitored with a thermo‐hygrometer; RS Components Handelsges GmbH, Gmünd, Austria) were within the range of 24°C and 50%, respectively. Mass of the micro test tube and sample was then taken eight to 10 times at 1‐min intervals with an analytical balance, whereby tubes were re‐placed under the light source between weighing. The rate of mass loss (representing water flow) showed no decrease during the measurement time, indicating approximately steady‐state flow (Sack *et al*., 2002). After the last measurement, the sample was removed from the micro test tube and equilibrated in a nylon bag with wet paper towels for 20 min. Plant water potential (Ψ_plant_) was then determined on leaf samples with C52‐psychrometers (using the same approach as described in ‘Pressure–volume analysis’ in Materials and Methods section) and LA was determined. The mean difference in mass *F* (mmol s^−1^) across time intervals was then divided by Ψ_plant_ to obtain absolute hydraulic conductance of shoots (*K*_shoot_; mmol s^−1^ MPa^−1^) and further by LA to obtain LA‐specific *K*_shoot_ (*K*_shoot_L_; mmol s^−1^ MPa^−1^ m^−2^). *K*_shoot_ values were then averaged for each species and age class. Xylem area‐specific shoot hydraulic conductance (*K*_shoot_Axyl_ *;* mmol s^−1^ MPa^−1^ m^−2^) was obtained by dividing mean *K*_shoot_ by mean xylem area (*A*_xyl_; see ‘Xylem anatomy’ in Materials and Methods section) for each species and age class. Standard errors for *K*_shoot_Axyl_ were estimated using a propagation of error formula based on the SE of *K*_shoot_ and *A*_xyl_ (Dunlap & Silver, 1986; Beikircher *et al*., 2021).

### Embolism resistance

Embolism resistance of hypocotyls was estimated by recording ultrasonic acoustic emissions (UAE; Nolf *et al*., 2015; Nolf *et al*., 2016) during bench‐top dehydration of four to 15 (for details, see Supporting Information Table S1) individuals per species and age class. A 150 kHz preamplified resonance sensor (PK151; Wolfegg, Physical Acoustic Corp., Germany) was attached in the centre of hypocotyls (Fig. 1) using a small spring clamp. Between hypocotyl and sensor, silicon grease was used to improve acoustic coupling. Signals of ≥ 35 db amplitude were recorded using AEWin™ (v. E3.35; Physical Acoustics Corp.). To slow down transpiration and guarantee water potential equilibration across organs, seedlings were loosely covered with dark plastic bags. At intervals, needles or leaf discs, respectively, were sampled and Ψ_plant_ measured as described above. To obtain Ψ_plant_ at any given time, linear interpolations between each two consecutive measured potentials were computed (Fig. S1; also see Nolf *et al*., 2015). Recording was stopped as soon as UAE ceased and/or Ψ_plant_ was not longer measurable.

For each individual, acoustic emission activity (AEA) was calculated in 5‐min increments and plotted vs respective Ψ_plant_. Water potential at main peak of activity (Ψ_AEAmax_; MPa) was determined by fitting the Savitzky–Golay smoothing function (Savitzky & Golay, 1964) to emission rates (see Nolf *et al*., 2015). Individual values were then averaged per species and age class.

### Xylem anatomy

Xylem anatomical analyses were carried out on hypocotyls of the same seedlings used for *K*_shoot_ measurements. Hypocotyls were preserved in an ethanol–glycerol–water solution (1 : 1 : 1, v/v/v) for several weeks. For each species and age class, transverse sections were cut with a microtome (Sledge Microtome GSL 1; Schenkung Dapples, Zurich, Switzerland) from the central part of three randomly chosen hypocotyls (Fig. 1), and stained for lignin with phloroglucinol–hydrochloric acid. Anatomical parameters were analysed with a light microscope (Olympus BX 41; System Microscope, Olympus Austria, Vienna, Austria) interfaced with a digital microscope camera (ProgRes CT3; Jenoptik, Jena, Germany), and with an image analysis software (imagej v.1.37; National Institutes of Health (NIH), Bethesda, MD, USA). Observations were made at a magnification of ×4 in a field of view of 0.55 mm^2^ for xylem area, and ×20 and 0.11 mm^2^ for conduit diameters and cell wall reinforcement, respectively.

Xylem area (*A*_xyl_) was calculated for each transverse section from pith and xylem diameters, directly measured on four positions (horizontal, vertical and twice diagonal), using the formula for the area of an annulus:

(Eqn 1) $$A_{\mathrm{xyl}}=\left(\pi /4\right)\times \left({d^{2}}_{X+P}–{d^{2}}_{P}\right)$$

where *d*_*X*+*P*_ is the diameter of the disc including pith and xylem and *d*_*P*_ that of pith alone (Beikircher *et al*., 2021).

From a total of 74 (3‐wk‐old *Pinus*) to 3582 (22‐wk‐old *Acer*) individually measured xylem conduit lumen areas per species, diameters (*d*; μm) were calculated assuming square shapes for conifers and circular shapes for angiosperms (Tomasella *et al*., 2018). To avoid unbalanced statistical weighting of samples with larger numbers of analysed conduits, diameters were first averaged for each individual and then for each species and age class to obtain mean diameter (*d*_mean_; Beikircher *et al*., 2021). Mean hydraulic diameter (*d*_h_) was calculated according to Sperry *et al*. (1994):

(Eqn 2) $$d_{h}=\sum d^{5}/\sum d^{4}$$

To determine cell wall reinforcement ((*t*/*b*)^2^; Hacke *et al*., 2001), for each hypocotyl transverse section, the double wall thickness (*t*) and conduit diameter (*b*) of the largest conduit within 13 (4‐wk‐old *Viburnum*) to 137 (22‐wk‐old *Acer*) conduit groups were directly measured. As for *d*_mean_, *d*_h_ and (*t*/*b*)^2^ were also first calculated per individual and then averaged per species and age class.

The Huber value (HV; i.e. the ratio of sapwood to LA; HV; m^2^ m^−2^) was calculated by dividing the mean *A*_xyl_ by the mean LA for each group. Standard errors were estimated using a propagation of error formula as for *K*_shoot_Axyl_ (see ‘Shoot hydraulic conductance’ in Materials and Methods section).

### Statistics

All datasets except for HV and *K*_shoot_Axyl_ were first tested for normal distribution and variance (Shapiro and the Levene test), and then, an ANOVA was performed using the Tukey (equal variance) or the Games–Howell (unequal variance) test to assess significant intraspecific differences across age classes. All tests were made at a probability level of 5% using R (v.4.2.2; packages agricolae and rstatix). As HV and *K*_shoot_Axyl_ were calculated based on mean values and SE estimated using a propagation of error formula (see respective sections above) differences across age classes were tested with a Welch test (*P* < 0.05; Rasch *et al*., 2011, Beikircher *et al*., 2021). Linear regression analyses (Pearson product–moment coefficient) were done with spss (v.27).

## Results

### Morphology

Three to 4 wk after germination, all species consisted of an hypocotyl and cotyledons. Conifers were on average significantly taller (3.08 ± 0.33 cm) compared with angiosperms (2.48 ± 0.60 cm), but had significantly lower projected LA (LA; 0.57 ± 0.07 vs 4.40 ± 3.52 cm^2^) and xylem area (*A*_xyl_; 0.005 ± 0.002 vs 0.115 ± 0.094 mm^2^; Table 1). By contrast, up to 29 wk later, at the end of the growing season, angiosperm species were overall taller with still significantly higher LA and *A*_xyl_ compared with conifers, interspecific differences though were high (Fig. 1). For instance, *L. decidua* was fivefold taller with threefold higher *A*_xyl_ and sevenfold higher LA than *P. abies*, while *A. pseudoplatanus*, which developed up to 18 true leaves during summer, was threefold taller, had sixfold higher LA and up to ninefold higher *A*_xyl_ than *F. sylvatica* and *V. lantana*, which only developed a maximum of eight true leaves (Table 1; Fig. 1).

Similarly, HV varied considerably between species, diversion and across different age classes within a given species. In conifers, HV increased on average from 0.92 ± 0.33 to 6.61 ± 2.32 during establishment, with the lowest values in *L. decidua*. In angiosperms, HV constantly increased in *V. lantana*, while it decreased in *A. pseudoplatanus*. In *F. sylvatica* it first decreased between 4 and 9 wk upon germination and then increased again. This was related to an initial significant increase in LA which then remained stable, while *A*_xyl_ further increased (Table 1).

### Cell osmotic parameters

**Fig. 2:**
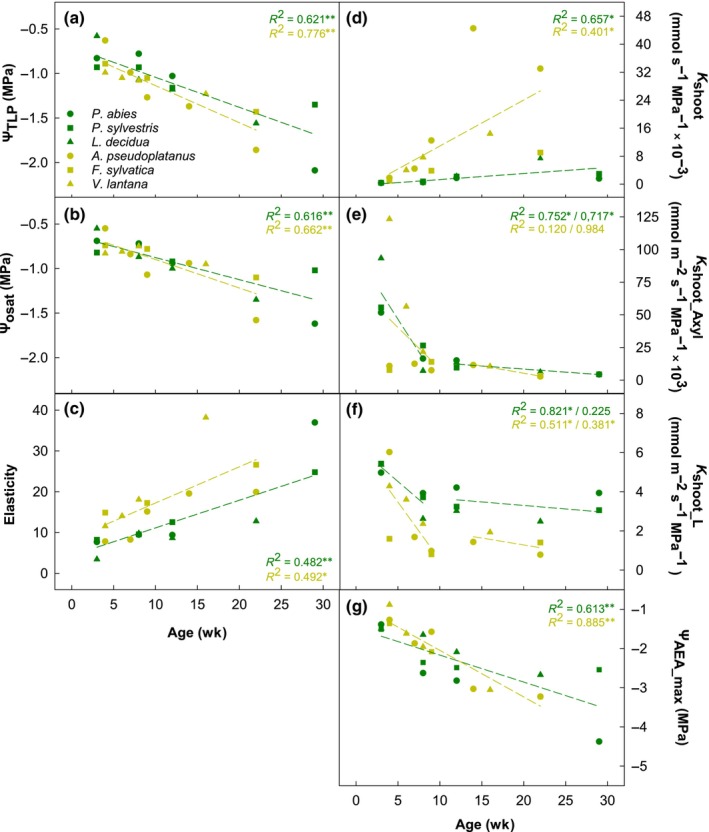
Ontogenetic changes in cell osmotic parameters, hydraulic conductance and embolism resistance. (a) Water potential at turgor loss (Ψ_TLP_), (b) osmotic potential at saturation (Ψ_osat_), (c) modulus of cell wall elasticity (ε), (d) absolute (*K*_shoot_), (e) xylem area‐specific (*K*_shoot_Axyl_) and (f) leaf area‐specific (*K*_shoot_L_) shoot hydraulic conductance as well as (g) water potential at maximum acoustic emission activity (Ψ_AEAmax_) of conifers (dark green) and angiosperms (light green) during seedling development. Lines show linear regressions for each lineage either across the whole time (a–d, g) or split into before and after 10 wk after emergence (e, f). Asterisks indicate respective statistical significance (*, *P* < 0.05; **, *P* < 0.01).

In the youngest age classes of both conifers and angiosperms, turgor loss occurred on average at −0.78 ± 0.10 MPa and −0.84 ± 0.11 MPa, respectively, and decreased to *c*. −1.6 MPa at the end of the growing season (Fig. 2a; Table S1). Although the values for the youngest and oldest stages were remarkably constant across species (0.4 and 0.8 MPa difference, respectively), the relative decrease within species varied considerably: in *P. abies* and *A. pseudoplatanus*, Ψ_TLP_ decreased by 1.2 MPa while in *V. lantana* no significant differences were observed between the first and last measurements. Osmotic potential at saturation (Ψ_osat_) showed a similar pattern. In conifers, as well as in angiosperms Ψ_osat_ decreased from *c*. −0.7 to −1.25 MPa, and seasonal changes were again most pronounced in *P. abies* and *A. pseudoplatanus* but negligible in *V. lantana* (Fig. 2b; Table S1). Initially, the modulus of elasticity (ε) was significantly lower in conifers (6.4 ± 1.5) than in angiosperms (11.4 ± 2.05). In all species, it increased significantly over the first growing season, indicating the presence of less elastic cells in older age classes. At the end of the growing season, no lineage‐related differences were evident. Again, changes within a given species differed considerably. For instance, ε increased fivefold in *P. abies* but only twofold in *F. sylvatica* (Fig. 2c; Table S1).

### Hydraulic conductance

On average, the absolute shoot hydraulic conductance (*K*_shoot_) was 0.33 ± 0.03 and 1.29 ± 0.30 mmol s^−1^ MPa^−1^ × 10^−3^ in the lowest age classes of conifers and angiosperms, respectively. In all species, it significantly increased over the season, but autumn values strongly differed across species and between lineages (Fig. 2d; Table S1): both, absolute values and relative increases were smaller in conifers, with the lowest values observed in *P. abies* (1.57 ± 0.33 mmol s^−1^ MPa^−1^ × 10^−3^). By contrast, *K*_shoot_ increased 18‐fold in *A. pseudoplatanus* with an absolute maximum of 33 ± 3 mmol s^−1^ MPa^−1^ × 10^−3^. *K*_shoot_ normalized by *A*_xyl_ (*K*_shoot_Axyl_) strongly decreased during development in all species (Fig. 2e). In conifers, *K*_shoot_Axyl_ decreased on average from 67 ± 13 to 5 ± 1 mmol m^−2^ s^−1^ MPa^−1^ × 10^3^, with the strongest decrease in *L. decidua*. In angiosperms, values differed significantly among species. While in *A. pseudoplatanus* and *F. sylvatica*, *K*_shoot_Axyl_ decreased from *c*. 9 to 3 mmol m^−2^ s^−1^ MPa^−1^ × 10^3^, in *V. lantana* it decreased 11‐fold from 123 to 11 mmol m^−2^ s^−1^ MPa^−1^ × 10^3^ (Table S1). In all species but *A. pseudoplatanus* and *F. sylvatica*, *K*_shoot_Axyl_ sharply decreased until Week 10 and then remained more or less stable until the end of the vegetation period. In *A. pseudoplatanus*, the decrease was less pronounced, while in *F. sylvatica*, values doubled until Week 10, and only then decreased (Table S1). A similar pattern was observed for *K*_shoot_ normalized by LA (*K*_shoot_L_; Fig. 2f). Initial values ranged between 4.28 and 6.02 mmol m^−2^ s^−1^ MPa^−1^, except for *F. sylvatica* with significantly lower values (1.60 mmol m^−2^ s^−1^ MPa^−1^; Table S1). In all species, *K*_shoot_L_ decreased during the first growing season, with the strongest changes in *A. pseudoplatanus* and least in *F. sylvatica*, and with a stronger decrease before Week 10 and lower thereafter.

### Embolism resistance

In 3‐ to 4‐wk‐old seedlings, main peak of AEA (Ψ_AEAmax_) was detected at −1.46 ± 0.04 MPa in conifers, and at −1.17 ± 0.15 MPa in angiosperms. Until the end of the growing season, Ψ_AEAmax_ significantly decreased in all species (Fig. 2g). In autumn, it was on average −3.23 ± 0.10 MPa in angiosperms, with the most pronounced decreases observed in *V. lantana*. In *P. sylvestris* and *L. decidua*, Ψ_AEAmax_ decreased to *c*. −2.6 MPa, but the most pronounced decrease, which also reached the lowest value, was observed in *P. abies* (−1.39 ± 0.08 to −4.38 ± 0.22 MPa; Table S1).

### Anatomy

Mean (*d*_mean_), maximum (*d*_max_) and mean hydraulic (*d*_h_) conduit diameters varied considerably across species, but no clear pattern across season was observed. Overall, all three parameters were higher in angiosperms than in conifers and changes were more pronounced in older age classes. For instance, compared with conifers, angiosperms had *c*. 1.7‐fold and 2.6‐fold higher *d*_h_ and *d*_max_ in spring and autumn, respectively. While *d*_max_ and *d*_h_ increased significantly from the first to last measurement in all species but *P. abies* an *P. sylvestris*, *d*_mean_ was remarkably similar across age. Only in *P. abies*, it decreased significantly and in *V. lantana* it increased (Table 2).

**Table 2:** Mean (*d*_mean_), mean hydraulic (*d*_h_) and maximum (*d*_max_) conduit diameter as well as cell wall reinforcement ((*t*/*b*)^2^) of investigated species and age classes.

Species and analysed age classes (weeks)	*d*_mean_ (μm)	*d*_h_ (μm)	*d*_max_ (μm)	(*t*/*b*)^2^
*Picea abies*				
3	7.9 ± 0.5^a^	9.5 ± 0.5^a^	10.9 ± 0.4^a^	0.025 ± 0.001^a^
8	6.8 ± 0.3^b^	8.6 ± 0.6^ab^	11.5 ± 0.9^a^	0.098 ± 0.010^ab^
12	6.8 ± 0.4^b^	8.9 ± 0.5^ab^	11.6 ± 0.8^a^	0.104 ± 0.002^b^
29	5.2 ± 0.1^c^	7.9 ± 0.7^b^	11.0 ± 1.1^a^	0.377 ± 0.038^c^
*Pinus sylvestris*				
3	7.8 ± 0.3^a^	9.0 ± 0.3^a^	9.2 ± 0.6^a^	0.057 ± 0.001^a^
8	7.8 ± 0.1^a^	9.4 ± 0.2^a^	12.7 ± 0.3^b^	0.098 ± 0.016^b^
12	7.7 ± 0.1^a^	9.4 ± 0.1^a^	12.0 ± 0.2^b^	0.106 ± 0.003^b^
29	7.7 ± 0.1^a^	9.9 ± 0.4^a^	12.9 ± 0.8^b^	0.116 ± 0.005^b^
*Larix decidua*				
3	5.8 ± 0.6^a^	6.8 ± 0.6^a^	8.6 ± 0.8^a^	0.069 ± 0.014^a^
8	7.5 ± 0.1^a^	9.2 ± 0.6^b^	11.8 ± 0.9^b^	0.133 ± 0.018^b^
12	6.3 ± 0.4^a^	8.0 ± 0.3^ab^	10.2 ± 0.4a^b^	0.137 ± 0.021^b^
22	6.6 ± 0.7^a^	8.8 ± 0.6^ab^	11.9 ± 0.8^b^	0.141 ± 0.015^b^
*Acer pseudoplatanus*				
4	10.7 ± 0.2^a^	16.4 ± 1.2^a^	21.7 ± 1.4^a^	0.014 ± 0.001^a^
7	9.4 ± 0.2^a^	16.3 ± 1.3^a^	21.4 ± 1.8^a^	0.014 ± 0.002^a^
14	10.7 ± 0.6^a^	19.9 ± 0.6^ab^	31.7 ± 1.1^b^	0.017 ± 0.002^a^
22	10.3 ± 0.5^a^	23.0 ± 1.7^b^	35.5 ± 1.3^b^	0.019 ± 0.000^a^
*Fagus sylvatica*				
4	6.9 ± 0.1^a^	18.5 ± 1.3^a^	22.2 ± 1.4^a^	0.012 ± 0.000^a^
9	6.4 ± 0.3^a^	19.4 ± 0.4^a^	23.6 ± 0.3^ab^	0.014 ± 0.002^a^
22	7.3 ± 0.2^a^	24.0 ± 1.6^b^	29.0 ± 1.7^b^	0.015 ± 0.001^a^
*Viburnum lantana*				
4	5.7 ± 0.3^a^	6.6 ± 0.6^a^	7.9 ± 0.9^a^	0.113 ± 0.010^a^
6	7.1 ± 0.6^ab^	13.5 ± 1.9^b^	17.2 ± 3.5^b^	0.018 ± 0.002^b^
8	7.2 ± 0.5^ab^	17.0 ± 0.8^bc^	20.3 ± 1.7^bc^	0.011 ± 0.002^b^
16	8.1 ± 0.3^b^	22.2 ± 1.9^c^	26.6 ± 1.8^c^	0.006 ± 0.001^b^

Mean ± SE. Different letters indicate significant differences (*P* < 0.05) across age classes within a given species.

Cell wall reinforcement ((*t*/*b*)^2^) increased significantly from 0.050 ± 0.009 in the youngest age classes of conifers to 0.212 ± 0.059 in the oldest classes (Table 2). In *A. pseudoplatanus* and *F. sylvatica*, (*t*/*b*)^2^ ranged from 0.012 to 0.019 with only slight increases during development. By contrast, in the shrub *V. lantana*, (*t*/*b*)^2^ decreased almost 19‐fold, whereby the high initial value can be attributed to few but thick conduits in 4‐wk‐old plants.

## Discussion

In course of the first growing season, tree and shrub seedlings undergo significant morphological, anatomical and therewith related hydraulic changes. Here, we show that independent of lineage, species and growth forms those changes are tightly coordinated, and across season the functional priority shifts from growth to maintenance. In the following, we discuss the observed findings and related ecological implications.

### Hydraulic conductance across the first growing season: from exceptional to stable supply

Driven by the increase in LA (larger LA allowing for higher transpiration) and enabled by an increase in xylem area (*A*_xyl_; more parallel paths allowing for larger water movements; Becker *et al*., 1999), shoot hydraulic conductance (*K*_shoot_) significantly increased over the first growing season in all species under study (Fig. 2d; Table S1). Slight decreases towards the end of the season were most likely related to the formation of latewood conduits and, in angiosperms, might also be related to embolism in larger conduits in late summer and autumn. However, the increase was not sufficient to maintain the high initial hydraulic sufficiency (i.e. leaf‐specific hydraulic conductance), as *K*_shoot_L_ sharply decreased over the first 10 wk and then remained more or less stable until the end of the first growing season (Fig. 2f; Table S1). This is consistent with previous observations (see Introduction section) and thus seems to be a general trend in seedlings of temperate woody plants. This also makes sense from an ecological point of view, as an optimal water supply to cotyledons and newly developed leaves is crucial to guarantee sufficient photosynthetic carbon synthesis in plants characterized by limited carbon storage (seed resources) and basically no water storage capacity (Losso *et al*., 2018). Previous studies have shown that limiting photosynthesis of cotyledons and newly developed leaves by, for example artificial inhibition of cotyledon photosynthesis, low‐temperature photoinhibition or shade, negatively affects seedling establishment and increases their mortality (Kozlowski, 1992; Germino & Smith, 1999; Germino *et al*., 2002; Facelli, 2008; Johnson *et al*., 2011). Although not yet proven, it can be assumed that the same is true for limiting water supply.

Given that in germinating seedlings the xylem is only starting to form, high hydraulic sufficiency can only be achieved by either a small LA and/or by highly conductive primary xylem elements. Indeed, in all conifer species, as well as in the shrub *V. lantana*, hydraulic efficiency (i.e. xylem area‐specific shoot conductance; *K*_shoot_Axyl_) was several‐fold higher in youngest stages despite a very small *A*_xyl_ (Tables 1, S1). In these species, the xylem consisted mainly of conducting elements, that were still arranged in bundles (Fig. 1). With the formation of the secondary xylem, *K*_shoot_Axyl_ strongly decreased and then, similarly to *K*_shoot_L_, remained stable until the end of the growing season (Fig. 2e). This is in line with the study of Miller & Johnson (2017) who also found the primary xylem to be more hydraulically conductive than secondary xylem. In *A. pseudoplatanus* and *F. sylvatica*, the first measured *K*_shoot_Axyl_ values were lower than in the other species, which can be explained by the already more advanced xylem development in these species: in 4‐wk‐old *Acer* and *Fagus* seedlings, a complete xylem ring with conducting and nonconducting elements had already formed (Fig. 1). We assume that an analysis of younger seedlings would have shown higher *K*_shoot_Axyl_ and, accordingly, a decrease in the following weeks, like in the other species. Interestingly, the high conductance of primary elements could not be directly related to conduit diameter. Although significant correlations of *d*_max_ and *d*_h_ with *K*_shoot_Axyl_ were found across all species and partly within lineages (Table 3), at the species level, significant changes with ontogeny were mainly observed in angiosperms and only for *d*_max_ and *d*_h_ (Table 2). Hence, high conductance is more likely related to pit characteristics (e.g. aperture fraction, pit chamber depth, pit membrane thickness, pit and pore size) that reduce flow resistance in the primary xylem (Choat *et al*., 2008; Lens *et al*., 2011; Schulte *et al*., 2015), and/or to an ionic effect (Nardini *et al*., 2012). Both, though, have yet to be investigated.

**Table 3:** Pearson correlations among anatomical, cell osmotic and hydraulic traits for all species and for each lineage treated separately.

	All species	Angiosperms	Conifers
*Correlations with K*_shoot_Axyl_			
*d*_mean_	−0.354	−0.538	−0.046
*d*_max_	−0.465*	−0.823**	−0.768**
*d*_h_	−0.440*	−0.892**	−0.443
(*t*/*b*)^2^	−0.057	0.915**	−0.511
*Correlations with* ψ_AEAmax_			
*d*_mean_	−0.010	−0.386	0.535
*d*_max_	−0.320	−0.873**	−0.375
*d*_h_	−0.240	−0.852**	0.114
(*t*/*b*)^2^	−0.448*	0.478	−0.866**
ψ_TLP_	0.805**	0.835**	0.809**
ψ_osat_	0.769**	0.726*	0.806**
ε	−0.718**	−0.747**	−0.827**
*K*_shoot_Axyl_	0.600**	0.598	0.621*

Xylem area‐specific shoot hydraulic conductance (*K*_shoot_Axyl_), mean (*d*_mean_), maximum (*d*_max_) and mean hydraulic diameter (*d*_h_), cell wall reinforcement ((*t*/*b*)^2^), water potential at main peak of acoustic emission activity (Ψ_AEAmax_) and at turgor loss (Ψ_TLP_), osmotic potential at full saturation (Ψ_osat_) and cell wall elasticity (ε).

*, *P* < 0.05; **, *P* < 0.01.

Notably, initial *K*_shoot_L_ was remarkably similar across lineages and species. Only in *F. sylvatica*, with its large cotyledons (Fig. 1), it was significantly lower than in the other species. This indicates that at least in their first weeks, angiosperm and conifer leaves are similarly well supplied with water. However, across the whole season, angiosperms had lower *K*_shoot_L_ than conifers despite higher *K*_shoot_ and similar *K*_shoot_Axyl_ (Fig. 2d–f; Table S1). This is mainly due to the higher ratio of xylem to LA in conifers and in older age classes. In conifers, the HV increased up to 10‐fold (*P. abies*) with age (Table 1). Our findings thus support previous studies showing that neither low growth rates nor low hydraulic capacity are typical traits of conifers (Becker *et al*., 1999; Becker, 2000; Coomes *et al*., 2005; Brodribb *et al*., 2012; Piper *et al*., 2019). By contrast, due to the much lower hydraulic resistance of the torus‐margo pit membrane of conifers (Pittermann *et al*., 2005; Sperry *et al*., 2006; Choat *et al*., 2008; Brodribb *et al*., 2012) and the specific composition (only conducting elements and parenchyma), conifer xylem may promote an overall better water supply to the leaves.

### Acoustic activity analysis reveals changing embolism resistance during seedling development

While an optimal water supply to leaves certainly promotes growth and thus increases the likelihood of successful establishment, sufficient drought tolerance is also crucial to ensure survival. Hydraulic safety, hereafter also referred to as embolism resistance, is one of its key parameters. However, due to methodological problems, almost nothing has been known about the hydraulic safety of tree seedlings (see Introduction section). In the present study, we used UAE analyses to estimate embolism resistance in shoots of current‐year seedlings. This technique has been used for decades (Nardini *et al*., 2024), and particularly in case of conifers, several studies have shown good agreement with other techniques (Milburn & Johnson, 1966; Tyree & Dixon, 1983, 1986; Lo Gullo & Salleo, 1991; Cochard, 1992; Hacke & Sauter, 1996; Mayr & Rosner, 2011; Mayr *et al*., 2014). By contrast, analyses of embolism resistance with UAE can be difficult in angiosperms, because ultrasonic emissions often continue to increase even after a total loss of conductivity. This is likely caused by embolism formation in fibres, emissions from nonconducting cells and tissues, or emissions due to microfracturing of wood (see Nolf *et al*., 2015; Rosner, 2015). Indeed, we found an almost linear increase in acoustic events at low water potentials in all investigated angiosperm species, but also in the youngest stages of conifers with a high proportion of living cells (Figs 1, S1). However, following the suggestions of Nolf *et al*. (2015) and Lamacque *et al*. (2022), we calculated the water potential at the main peak of activity (Ψ_AEAmax_) as a measure of the water potential at maximum embolism formation and thus obtained stable and comparable results in all age classes and species. Furthermore, Ψ_AEAmax_ of the oldest stages was remarkably similar to the water potential at 50% loss of hydraulic conductance (P50) reported for branches and stems of mature trees and shrubs (Cochard, 1992; Beikircher & Mayr, 2009; Lens *et al*., 2011; Charra‐Vaskou *et al*., 2012; Choat *et al*., 2012; Li *et al*., 2016). Thus, we deem this approach appropriate to estimate embolism resistance in tree seedlings.

**Fig. 3:**
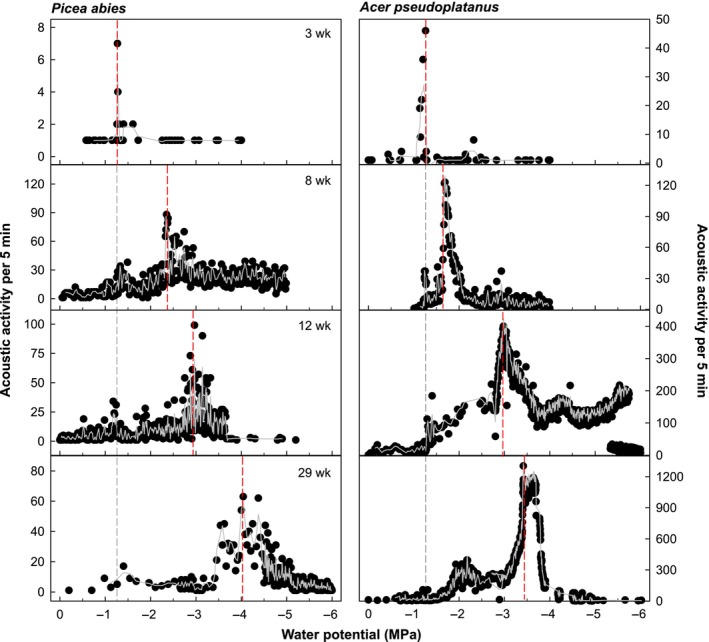
Acoustic activity vs water potential of differently aged *Picea abies* (left panels) and *Acer pseudoplatanus* (right panels) seedlings. Shown are 5‐min means (black circles) and Savitzky–Golay smoothed values (grey line). Vertical lines show water potential at main peak of acoustic activity (Ψ_AEAmax_) of the respective plant (red) and of the youngest investigated plants (black).

Based on these analyses, embolism resistance of the youngest stages was remarkably low in all study species, with Ψ_AEAmax_ ranging between −0.88 and −1.51 MPa. The embolism resistance significantly increased over the growing season in all species (Fig. 2g; Table S1). At the end of the growing season, it reached values comparable to P50 of older plants (described in the previous section) and, in case of *Acer* and *Fagus*, values in the range of P50 of 6‐month‐old seedlings obtained with classic hydraulic flow measurements (Losso *et al*., 2018). Notably, while Ψ_AEAmax_ significantly shifted towards lower water potentials during establishment, older seedlings often still exhibited additional small peaks at higher water potentials. The most pronounced of these peaks were at water potentials when Ψ_AEAmax_ occurred in the youngest stages (Fig. 3), which may be related to embolism formation in the more vulnerable primary conduits. Indeed, microCT analyses of 4‐ to 6‐wk‐old conifers revealed that primary xylem elements embolized at significantly higher (i.e. less negative) water potentials than secondary ones (Miller *et al*., 2020). The same was also observed in current‐year shoots of mature trees (Choat *et al*., 2005; Venturas *et al*., 2019), sapling stems (Choat *et al*., 2015) and herbaceous plants (Haverroth *et al*., 2024), and is most likely related to the particular wall structure of primary conduits, such as lower cell wall reinforcement and torus‐to‐pit overlap. Both parameters have repeatedly been connected to higher resistance to tracheid implosion and air seeding (Hacke *et al*., 2001; Domec *et al*., 2009; Hacke & Jansen, 2009; Bouche *et al*., 2014; Song *et al*., 2022). Similar to Miller & Johnson (2017) and Miller *et al*. (2020), we also observed a significant increase in cell wall reinforcement ((*t*/*b*)^2^) during ontogeny (Table 2) and a significant correlation with decreasing Ψ_AEAmax_ (Table 3) in all conifers. However, in angiosperm seedlings (*t*/*b*)^2^ increased only slightly in *Acer* and *Fagus* and even decreased in *Viburnum*, indicating that the decrease in Ψ_AEAmax_ was most likely related to other anatomical characteristics (e.g. pits, vessel length, conduit connectivity; Lens *et al*., 2011; Li *et al*., 2016; Mrad *et al*., 2021; Pritzkow *et al*., 2022). The decrease in *V. lantana* was particularly unexpected, but a similarly low (*t*/*b*)^2^ as found in the last stage was also reported by Beikircher & Mayr (2009) for adult plants of the same species, and may be related to the shrubby growth form. De Micco *et al*. (2008) also reported lower absolute vessel wall thickness and no increase with wood age in shrubs compared with trees. More detailed anatomical analyses on differently aged seedlings of varying species and growth forms would improve our knowledge on seedling anatomy and related hydraulics.

### Tight linkage between hydraulic safety and cell osmotic parameters

Parallel to hydraulic safety, the drought tolerance of living leaf tissues also increased during development: in all species except *Viburnum*, water potential at turgor loss point (Ψ_TLP_) and osmotic potential at full saturation (Ψ_osat_) were highest (i.e. least negative) in youngest stages and significantly decreased thereafter (Table 2). The Ψ_TLP_, mainly depending on Ψ_osat_, has been recognized as a reliable trait to estimate drought tolerance (Bartlett *et al*., 2012) as low Ψ_TLP_ enables plants to maintain gas exchange with decreasing soil and plant water potentials (Sack *et al*., 2003; Blackman, 2018). Feng *et al*. (2023) showed that even in mature plants neither Ψ_TLP_ nor embolism resistance are static traits but can shift in tight coordination during the growing season. It can be assumed that in seedlings, constant adjustments of these parameters to altered morphological, hydraulic and environmental conditions are even more important. Accordingly, we observed a strong correlation of Ψ_TLP_ as well as Ψ_osat_ with Ψ_AEAmax_ both across all species as well as within angiosperms and conifers (Table 3). Also, Alvarez‐Cansino *et al*. (2022) found a strong correlation between turgor loss point and drought survival for tropical seedlings.

In contrast to Ψ_osat_, the role of the modulus of elasticity (ε) on Ψ_TLP_ and drought tolerance in general is less clear. We found strong negative correlations between ε and Ψ_AEAmax_ (Table 3) indicating that higher stiffness (i.e. higher ε) correlates with higher embolism resistance (i.e. lower Ψ_AEAmax_). This contradicts the assumption that elastic walls are able to postpone turgor loss. However, Bartlett *et al*. (2012) also observed high ε in plants growing in dry biomes and an increase in droughted plants. The authors suggested that an increase in ε may act to maintain relative water content at turgor loss despite decreases in Ψ_osat_ required to obtain low Ψ_TLP_. Interestingly, our findings extend their observed correlations across species and biomes to developmental stages in seedlings.

We assume that the risk for low leaf water potentials increases with seedling age due to (1) the observed ontogenetic decoupling of xylem and leaf traits and consequently reduced hydraulic sufficiency (described in the previous section), (2) an increase in transpiration due to larger LA and at least in some species also an increase in stomatal conductance with leaf order as observed by Beikircher *et al*. (2021) for *Acer* seedlings, and (3) a general increase in air temperatures and VPD as well as a decrease in soil moistures towards midsummer in temperate regions. Thus, a decrease in Ψ_TLP_ combined with an increase in hydraulic safety might be crucial for seedlings to survive beyond the first summer, although these parameters are only some aspects of plant drought tolerance. With limited water and carbon storage, seedlings may still be more prone to drought‐related mortality than juvenile or adult trees with the same vulnerability thresholds or turgor loss points.

### Conclusions and outlook

**Fig. 4:**
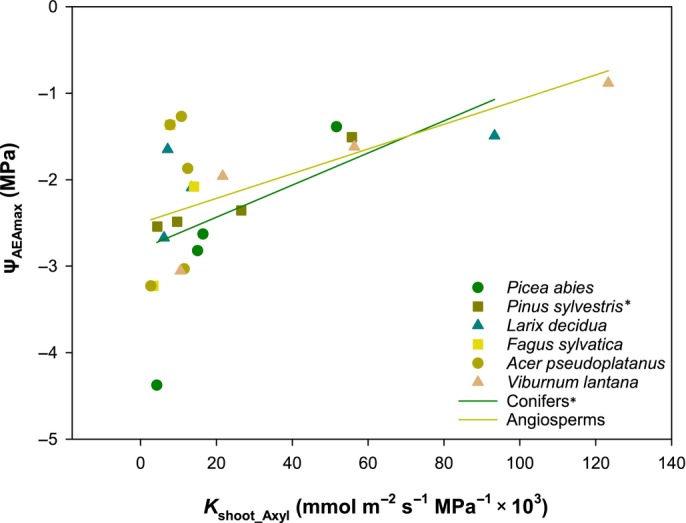
Relationship between water potential at maximum acoustic emission activity (Ψ_AEAmax_) and xylem area‐specific shoot conductance (*K*_shoot_Axyl_) for seedlings of different species (symbols) and lineages (lines). Asterisks indicate statistically significant correlations for a given species or group, respectively (*, *P* < 0.05).

The surprisingly consistent trend in the development of hydraulic conductance and safety across lineages and growth forms suggests some general underlying requirements. Based on our findings, we propose that the functional priority of the overall well‐coordinated morphological, anatomical and hydraulic changes shifts during tree seedling development: in newly establishing seedlings, maximizing photosynthesis is crucial to become independent of seed reserves and to allow initial growth for optimal access to soil water‐ and light resources (Tyree, 2003; Fenner & Thompson, 2005; Leck *et al*., 2008; Augustine & Reinhardt, 2019). At this stage, restrictions in order to achieve higher safety might even be fatal if carbon uptake is impeded. Later though maintenance of a stable water supply may become more important and, given the increasing risk of drought as the season progresses in temperate regions, hydraulic safety needs to be adjusted. Notably, such a shift may be influenced by an efficiency–safety trade‐off, which has been controversially discussed in recent years (Gleason *et al*., 2016). In our dataset, we found a strong correlation between *K*_shoot_Axyl_ and Ψ_AEAmax_ across all species and age classes, but within lineage correlations were only significant for conifers, and within species only for *P. sylvestris* (Table 3; Fig. 4).

Altogether, our findings strongly support the hypothesis that cotyledons and first leaves of temperate tree and shrub seedlings are exceptionally well supplied with water to promote successful seedling establishment, while well‐coordinated morphological, anatomical and hydraulic changes enable stable hydraulics in older seedling stages. In view of climate change, further studies are needed to expand our knowledge of the hydraulic safety and drought tolerance of seedlings. In addition to identifying key parameters of drought tolerance, it will also be important to recognize the influence of various parameters ranging from seed origin (e.g. population and year of seed maturation), to the influence of environmental conditions (e.g. late frosts, spring droughts, irrigation and soil properties) as well as the acclimation potential of seedlings. Ultimately, such data provide the basis for reliable predictions of the natural regeneration capability of forests as well as for future restoration projects.

## Competing interests

None declared.

## Author contributions

BB and SM planned and designed the present study. BB performed hydraulic experiments and MH anatomical analyses. BB analysed the data and prepared the manuscript with guidance from SM. AL and MH provided input on the final version of the manuscript.

## Supporting information


**Fig. S1** Representative graphs showing cumulative acoustic emission and acoustic activity with decreasing water potential.

**Table S1** Hydraulic efficiency and safety as well as cell osmotic parameters of investigated species and age classes.
Please note: Wiley is not responsible for the content or functionality of any Supporting Information supplied by the authors. Any queries (other than missing material) should be directed to the *New Phytologist* Central Office.

## Data Availability

Data supporting this study but not presented in the publication are openly available in the University of Innsbruck repository at doi: 10.48323/zgj2n‐f3644.
