# Supplementary material for: New insights into a sensitive life stage: hydraulics of tree seedlings in their first growing season
Source: New Phytol. 2024 Nov 5;245(2):577–90. doi: 10.1111/nph.20243 (PMC11655438; doi:10.1111/nph.20243)
Supplement: Supplementary file 1 — Fig. S1 Representative graphs showing cumulative acoustic emission and acoustic activity with decreasing water potential. [file NPH-245-577-s002.docx]

## *New Phytologist* Supporting Information

Article title: New insights into a sensitive life stage: Hydraulics of tree seedlings in their first growing season

Authors: Barbara Beikircher, Magdalena Held, Adriano Losso, Stefan Mayr

Article acceptance date: 14 October 2024

The following Supporting Information is available for this article:

**Fig. S1** Representative graphs showing cumulative acoustic emission and acoustic activity with decreasing water potential.

**Table S1** Hydraulic efficiency and safety as well as cell osmotic parameters of investigated species and age classes.

**Fig. S1** Percentage cumulative hits (solid line) and percentage acoustic emission activity (open circles; left y-axes) as well as plant water potential (filled blue circles; right y-axes) *versus* time for (a) three- and (b) 29-week-old *Picea abies* and (c) four- and (d) 22-week-old *Acer pseudoplatanus* seedlings. Dashed vertical lines highlight main peak of acoustic emission activity (Ψ_AEAmax_), dotted lines show linearly interpolated water potentials.

**
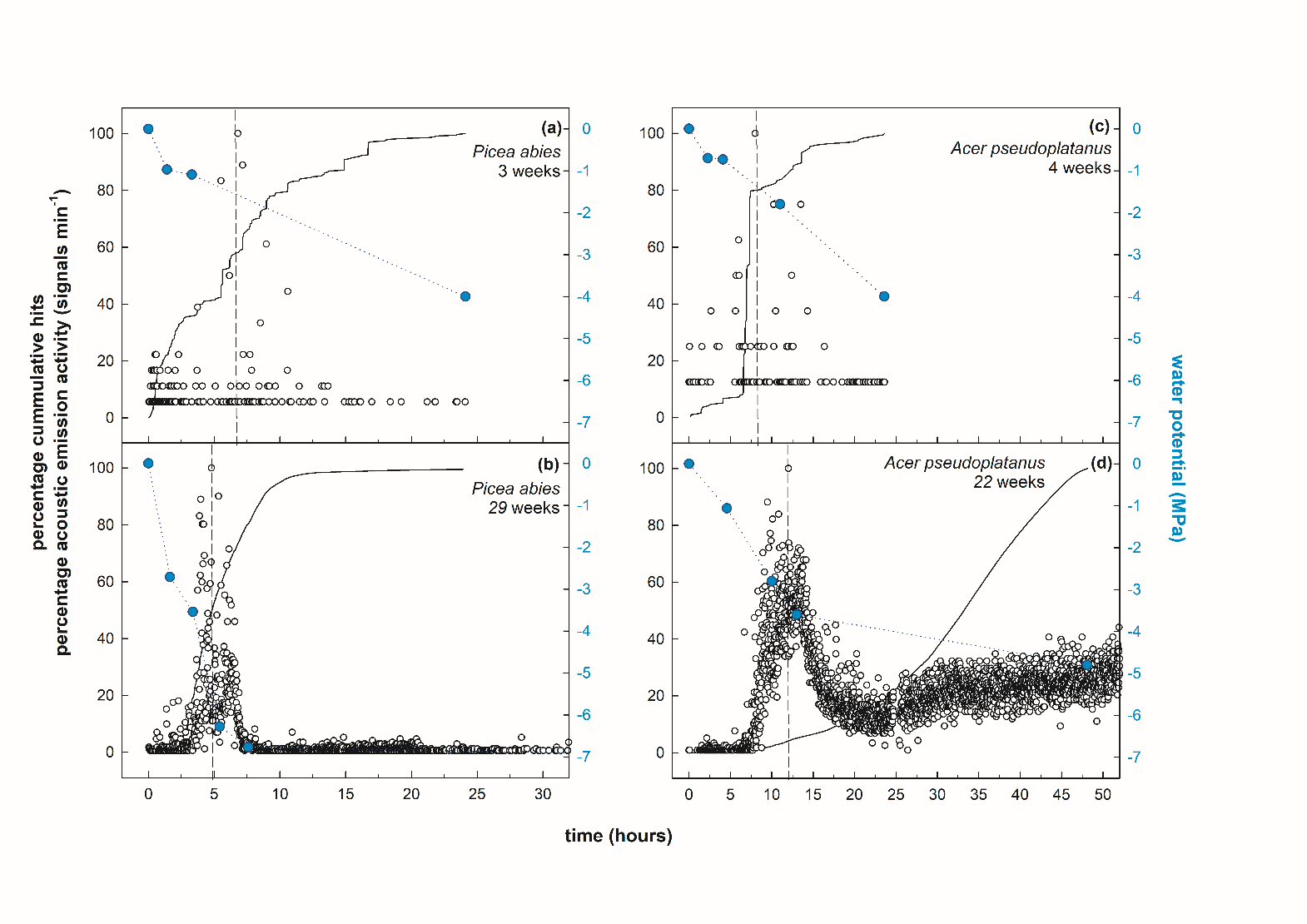
**

**Table**

Absolute (*K*_shoot_), xylem area- (*K*_shoot_Axyl_) and leaf area-specific (*K*_shoot_L_) shoot hydraulic conductance, and water potential at main peak of acoustic emission activity (Ψ_AEAmax_) as well as water potential at turgor loss (Ψ_TLP_), osmotic potential at saturation (Ψ_osat_) and modulus of cell wall elasticity (ε) of investigated species and age classes.

**Table uploaded as Excel file.**

**Footnote:** Mean ± SE. n shows number of plants used for conductance, ultrasonic and cell osmotic analyses, respectively. Different letters indicate significant differences (P<0.05) across age classes within a given species.
